# The complete mitochondrial genome of *Neolissochilus stracheyi* (Osteichthyes:Cyprinidae)

**DOI:** 10.1080/23802359.2022.2107460

**Published:** 2022-08-17

**Authors:** Zhenzhu Wei, Bo Zhao, Xinyan Lin, Chunyan Yin, Shihan Xu, Xiaoxin Dai

**Affiliations:** College of Fisheries, Zhejiang Ocean University, Zhoushan, China

**Keywords:** *Neolissochilus stracheyi*, phylogeny, mitochondrial genome

## Abstract

*Neolissochilus stracheyi* Day 1871 is a rare specie of fish inhabit clear forest streams and rivers. In order to discuss the phylogenetic position of *N. stracheyi*, the mitochondrial genome was obtained by sequencing. The genome was 16,587 bp in length with an accession number OM203155. The AT contents were 56.59%. The location and composition of genes are consistent with published Cyprinids containing 13 protein-coding genes, 22 transfer RNA genes, 2 ribosomal RNA genes, and 1 main non-coding regions. Sequence analysis showed that the mitochondrial genome of *N. stracheyi* has high sequence homology with other cyprinid fishes. Phylogenetic tree results showed that *N. stracheyi* is most closely related to *Neolissochilus heterostomus*. The mitochondrial sequence is of great significance for fish conservation, taxonomic status and resource exploitation.

## 1. Background

*Neolissochilus stracheyi* Day 1871 which belongs to Cypriniformes, Cyprinidae, *Neolissochilus*. The fish body color is bronze on the back and silver on the abdomen, with black side stripes on the body surface. In recent years, due to environmental pollution and other reasons, the *N. stracheyi* decreased sharply. *N. stracheyi* is a rare fish specie, and this fish is distributed in the valley of Daying River and Longchuan River in Yunnan Province, China, and it has rich nutritional value (Hei and Sarojnalini 2015). In order to provide useful information for the future research of genetic diversity and phylogenetic, we sequenced the whole mitochondrial genome of *N. stracheyi* and analyzed the main structural information of its genome (GenBank accession number OM203155).

## 2. Methods

*N. stracheyi* was obtained from a stream of the Dayingjiang River in Yunnan Provincein, China, in October 2021; 25.034058°N, 102.674134°E. Fish was identified according to morphological characteristics, the specimen was snap-frozen by liquid nitrogen and preserved under −80 °C and then transferred to laboratory, took the muscle from the back of the *N. stracheyi.* The rest tissues were deposited in the Biological Herbarium of Zhejiang Ocean University, with certificate number WPZ20211207, contact person (Wei Zhenzhu, 15503403858@163.com). The total genomic DNA was extracted from tissues using the DNA E.Z.N.A® tissue DNA kit (OMEGA, China).

The complete mitochondrial genome sequences of 14 species of Cyprinoidei were obtained from the GenBank database of NCBI. Sequences were assembled using GetOrganelle: (https://github.com/Kinggerm/GetOrganelle) (Jin et al. 2020). The phylogenetic trees were constructed by neighbor join (NJ) (Zhang and Sun 2008) and maximum likelihood (ML) (Pattengale et al. 2010) methods. The NJ trees were obtained by 10,000 bootstrap (Cuadra et al. 2020) copies using MEGA7.0.

## 3. Results

The complete mitochondrial genome of *N. stracheyi* is 16,587bp in length and consists of 13 protein-coding genes (PCGs), 22 transfer RNA genes (tRNA), 2 ribosomal RNA genes (rRNA) and 1 control regions (D-loop). The mitogenome base composition was A 31.81%, T 24.78%, C 15.74%, and G 27.67%, A + T content (56.59%) was a little more than the G + C content (43.41%) (Cheng et al. 2017) in common with other vertebrate mitogenomes. Except for 8 tRNA and the ND6 genes encoded on the L-strand, the other genes were encoded on the H-strand. Among the 13 PCGs of *N. stracheyi*, the starting codon of 12 PCGs were ATG, except COX1 gene which was GTG. Six PCGs (ND1, COX1, ATP6, ND5, ND4L and ND6) used TAA as the stop codon, and three PCGs (ND2, ATP8 and ND3) used TAG as the stop codon. Two complete stop codons (TAA, TAG) and two incomplete stop codons (TA-, T–), which can be completed by post-transcriptional polyadenylation, these incomplete termination codons can be accomplished by post-transcriptional polyadenylation and is generally present in the mitochondrial genome of teleost fish (Chu et al. 2013; Wang et al. 2016).

## 4. Discussion

**Figure 1.**
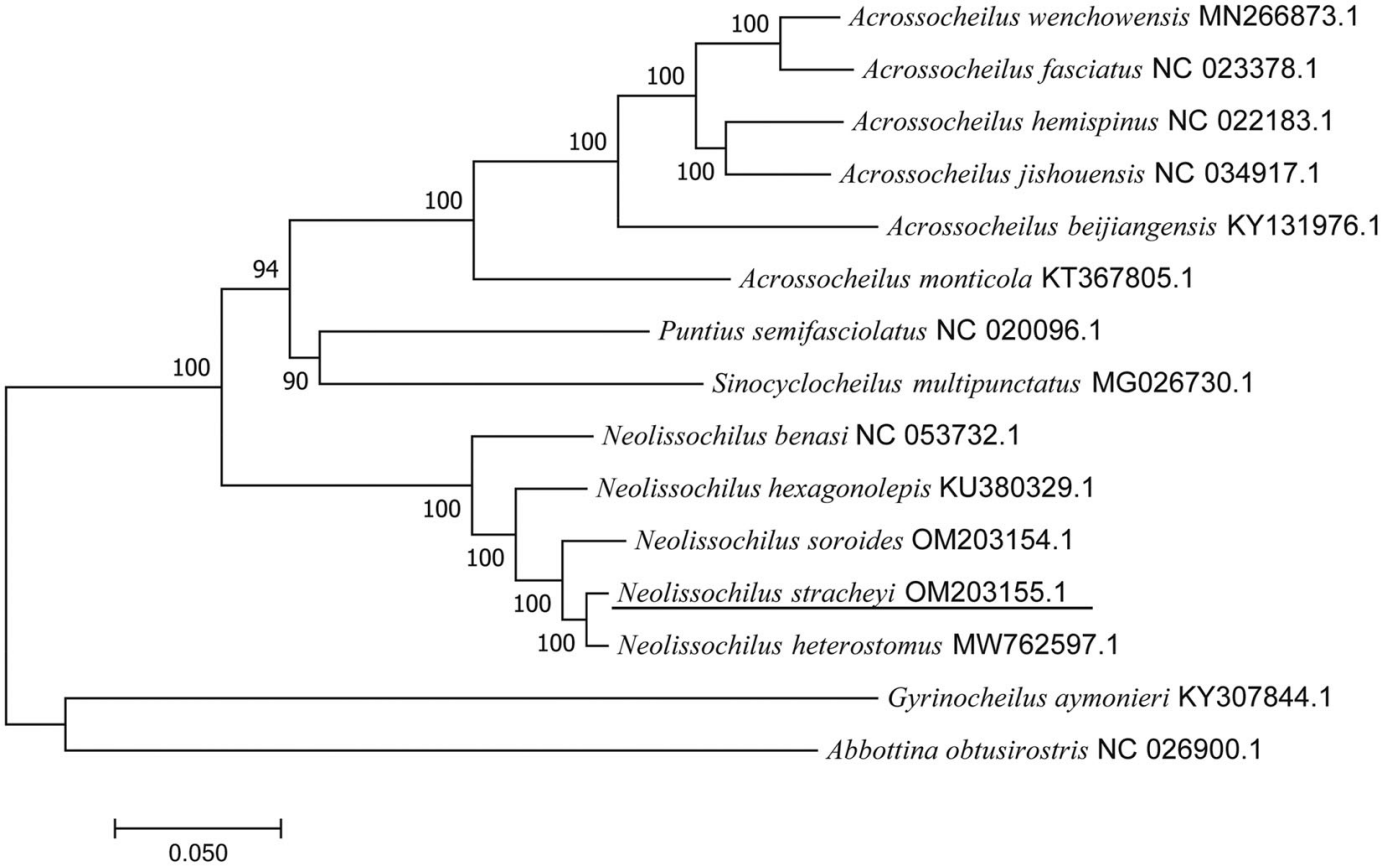
The phylogenetic tree is based on complete mitochondrial genomes of *N. stracheyi* and other 14 species.

A phylogenetic tree reconstructed by 15 complete mitochondrial genomes reveals that *N. stracheyi* is mostly related to *Neolissochilus heterostomus* (Figure 1). This mitochondrial information of *N. stracheyi* will benefit relative ecological and phylogenetic studies.

## Data Availability

The complete mitochondrial genome sequence of *Neolissochilus stracheyi* has been deposited in GenBank and openly available in GenBank of NCBI at https://www.ncbi.nlm.nih.gov/nuccore/OM203155 under the accession no. OM203155. The associated BioProject, SRA, and Bio-Sample numbers are PRJNA817097, SRR18356108, and SAMN26747789 respectively.
